# Modeling Compound vs. Plaster of Paris*We are reprinting this article because it contains a considerable measure of truth although expressed in critical form.

**Published:** 1916-12

**Authors:** Dayton Dunbar Campbell

**Affiliations:** Kansas City, Mo.


### MODELING COMPOUND vs. PLASTER OF PARIS.*

BY DAYTON DUNBAR CAMPBELL, D.D.S., KANSAS CITY, MO.

A cursory examination of our dental journals for the past few months will show that considerable interest has been revived in the Prosthetic branch of our profession, and especially along the lines of impression taking.

Most notable, perhaps, is the procedure or technique first outlined by Dr. P. G. C. Hunt, of Indiana, years

*We are reprinting this article because it contains a considerable measure of truth although expressed in critical form.

ago, and which, in the last decade, has been improved and revised, and so enthusiastically taught by the late Dr. J. W. Green, of Chillicothe, Mo.

Mr. Samuel L. Supplee, who conducts one of the finest and best laboratories in New York City, is our modern apostle and champion of the compound method and, being a man of charming personality and an enthusiastic lecturer, he is much in demand by those who would venture into the fields of Prosthesis. The writer is thoroughly familiar with the Green Method, and at the time Prof. Alfred Gysi, of Zurich, was in New York, had the very pleasant opportunity of reviewing the Green Method with Mr. Supplee, who was, at that time, associated with Dr. Gysi in teaching the most modern Prosthetic procedure. Mr. Supplee has always acknowledged his indebtedness to Dr. Green for the technique employed —except for one or two hair-splitting details—the importance of which was greatly magnified by the former and only suggested by the latter. Under this head must be mentioned specifically the taking of impressions "Under normal biting stress."

It is interesting to note that both men taught a technique and absolutely ignored nearly all of the fundamental principles and factors underlying the retention of artificial dentures, still the writer believes that no one man has done so much in the last twenty years to teach the art of impression taking as Dr. J. W. Green, but he also believes that through all the years of his teaching, there entered into his technique an *element of unconscious deception,* and that this element was the basis of his apparent success. We believe that its most loyal and enthusiastic advocate, our good friend Mr. Supplee, is laboring under the same delusion. We have asked literally dozens of dentists who have taken instructions in this method of manipulating compound if they, in any case in their practice, had constructed a denture that after being used awhile, even approximately compared in retention with the "test" impression. We have never received an answer in the affirmative.

Is this on account of faulty technique or a lack of attention to detail? No, the cause is directly traceable to this "element of unconscious deception." It is noteworthy that those men who are most familiar with denture construction from a practical, busy-practice standpoint are not at all enthusiastic about this modeling compound method.

Many dentists, after taking a course in the manipulation of modeling compound, have set themselves up as superior workmen in this line. and after taking an impression for the patient of another dentist, ask that their responsibility cease. Those enthusiastic disciples of Mr. Supplee would have you understand that "when an apparently good impression has been taken, and a plate made from it does not fit, the blame should be laid to the fault of the vulcanizer or to some stage of the procedure following the taking of the impression."

A very brief resume of the method appearing in the late issues of the various dental journals in the minutest detail, is as follows: An approximately well adapted tray of special design. Kerr Modeling Compound to be manipulated at 160 Fahr. After placing the compound in the tray, place the tray in the mouth. The excess over the labial and buccal borders of the tray is well massaged by pressure exerted upon the lips and cheeks. Chill and remove. The compound is then trimmed up sharp on the labial and buccal borders and the "very edge" heated slightly and quickly returned to the mouth. The patient, who has previously been coached in muscular gymnastics, now begins the facial contortions and this action causes the labial and buccal frenum to cut into the softened compound to a position of rest, et cetera. The posterior palatal portion of the compound is now softened and pressed well up against the vibrating (?) soft palate either by the action of the patient's tongue or the operator's forefinger. This seals the posterior portion of the palate and, after chilling, prevents the ingression of air.

Presto! See the vain attempts the operator makes to dislodge the test impression. Will he never succeed? Perhaps the patient can help. Whistle! Cough! Spit! Sneeze! Laugh! Cry! No! No! Don't be alarmed, my good man. It can be removed. I have the invisible key here between the thumb and forefinger of my right hand. By placing the thumb of the aforesaid right hand upon the posterior palatal portion of this impression and the forefinger of the self same hand over the posterior buccal border and in the direction of the malar process, the muscles are unlocked (?).* (Air is admitted and the vacuum destroyed.)

Still, such a prominent authority as Dr. George Henry Wilson, of Cleveland, Ohio, author of one of our best books on Dental Prosthetics, makes the following statement on page 57 of his second revised edition: "There is no legerdemain associated with prosthesis, the profession being only an expression of cause and effect; therefore, it behooves one to cultivate thoughtfulness and thoroughness in manipulation."

For the benefit of those who would like to try the experiment: When the impression is completed or has reached comparative perfection, it will be interesting to note that the muscle-trimmed, "valve-like" portion, through which the little "so-called muscles" cavort around in their various positions with "relief without leak," may be filled with compound and in no way will the "sticktion" or suction be impaired. This vacuum is the crux upon which the entire technique hangs. It constitutes the element of unconscious deception. When the lips and cheeks were massaged, there was created a closer peripheral adaptation. Then the posterior por-

---

**Dental Summary*, October, 1916. Page 817—"How to Use Muscles to Hold a Plate."

tion was pressed up until it fit snug against the soft tissue, which, in turn, dropped easily over the border in a not uncomfortable position. By forceful swallowing, the patient is enabled to almost completely expel the air from over the entire area of the impression. With the impression covering such a large area and the vacuum almost perfect, any effort to dislodge the plate must overcome the law of atmospheric pressure.

By the use of Spence Plaster Compound or Weinstein's Cast Material, it is impossible to construct a base or denture that will duplicate every "test" we give the above impression, but it is only a matter of a week or two until the tissues, in response to the laws of atmospheric pressure, are drawn down into the denture to occupy the former vacuum. Then it is you are greeted by the little old man, who gleefully demonstrates that he does not have to whistle, cough, spit, sneeze or blow to "get 'em" down.

Then to the uninitiated the process of "rerimming" begins. This procedure almost produces "the thrill that comes but once in a lifetime," but it is always accompanied by the same disastrous results, and for the reasons already explained.

Then the life work of Dr. J. W. Green has been fruitless? By no means. We have learned that the majority of dentures fail from one or more of the following reasons: First, the trays are off size and improperly adapted. Second, failure to get the impression high enough over the tuberosities. Third, using an over-size tray and permitting the plaster to drop at that point where it should be held tight against the tissues. Fourth, that the coaching and training of the patient to whistle, cough, spit, sneeze, blow, together with the facial gymnastics, is disastrous from a psychological standpoint, because in so doing we virtually assume all of the responsibility for a perfect adaption of the denture, which we assert by our actions, if not in words, is proof against any muscular contortion.

### IMPRESSIONS WITH THE MOUTH CLOSED AND UNDER NORMAL BITING STRESS.

The distribution of stress or pressure over the entire area of the supporting structure is of prime importance and is absolutely essential if the dentures are to be worn with the utmost comfort and serve in the most efficient manner. To expect that the equalized pressure obtained in taking the impression can be maintained through the subsequent procedure, and especially where casts of plaster and bolt flasks are used, is folly.

If, by the most careful technique and attention to details, the operator succeeds in securing three points of contact, porcelain to porcelain, he is justified in believing that his method of procedure has been comparatively faultless. Dr. Rupert E. Hall, formerly of Houston, Texas, advises that a thin sheet of yellow base-plate beeswax be placed upon the lower denture, covering the entire occlusal surfaces of all the teeth and a corrected bite be taken with both of the completed dentures in position. The dentures are then returned to the articulator sealed together and the new relation maintained in the old position by constructing a new cast for either the upper or lower. The teeth of the dentures are now automatically ground by placing a mixture of No. 90 grit carborundum powder and glycerine upon the antagonizing surfaces, and working the articulator to correspond to masticatory movements of the human mandible.

The writer takes great pleasure in stating that the above method of equalizing the normal biting stress has been the source of more satisfaction than any one particular ''stunt'' that he has acquired in several years.

### HALL METHOD OF PLASTER IMPRESSIONS.

Nearly three years ago Dr. Hall became dissatisfied with the vulcanite bases which he had obtained by using modeling compound in taking the impressions, and again turned to plaster.

His dissatisfaction with modeling compound had prevailed, despite his most studied attempts to follow in its use the directions given by Dr. Green in his clinics and so uniquely described in his text book. The very helpful and profusely illustrated book, "Prosthetic Articulation," could not supply him with the reason why the base, constructed upon an unchangeable cast, was not retained by the patient as well as the impression itself. Had the writer of this paper not experienced in obstinate cases exactly this same difficulty, and had he not known Dr. Hall personally, and been able through association with him to form an opinion as to his ability as an operator, this method, new to many, might have been allowed to escape without notice or comment.

The Hall Method of plaster impressions is applicable more especially to securing a corrected impression of the edentulous jaw. When time is a factor its employment is a distinct advantage. The fact that the writer has obtained by this method a greater per cent. of bases that did not require numerous alterations, is offered as a justification for this article.

The whole idea of muscle trimming, posterior palatal adaptation, and relief without leak—justly credited to Dr. Green, who deserves much praise for these and many other contributions to this branch of the prosthetic art —is recognized and incorporated in this method, which eliminates the vacuum.

The first step in the procedure is to select a large over-size tray and take an impression with modeling compound without any special attempt to secure detail. Remove the impression and place in cold water, when the tray may be removed from the compound without distortion. With a sharp knife trim away fearlessly the excess from heel to heel and well up onto the buccal and labial borders.

This improvised tray thus made of modeling compound is partially filled with impression plaster mixed to the proper consistency, placed into position in the mouth, and the cheeks and lips allowed to resume a position of rest. After a moment the tray may be released, and the plaster allowed to set hard, while the operator cleans the plaster bowl and spatula to be ready for the second mix. The first impression with plaster will reveal a number of places where the compound shows through. These places prevent an equal distribution of the pressure and they together with the surplus plaster at the line of demarkation between the hard and soft palate, must be cut away. The impression is now placed in water so that the plaster of paris may take up here the water of crystallization, which it otherwise would absorb from the next application of plaster—a result which would hinder a free manipulation.

A second and very thin mix of plaster preferably with warm water and salt (this depends, however, upon the individual operator's methods of manipulation) is now applied. Using a brush, a layer approximately an eighth of an inch thick is applied to the inner surface or intaglio of the impression, and this quickly inserted into position —and the patient cautioned *not* to move the lips or cheeks.

After a moment the tray is again released and preparations made for the next mix. When the small pieces of plaster in the mouth indicate, the impression is removed and examined. Any uncovered compound is an indication of pressure and is cut away, the posterior border is trimmed again, the excess of plaster beneath the compound tray removed, the impression coated again with thin plaster and the process described above repeated.

If the operator prefers, a sufficient amount of plaster may be mixed to allow the entire impression to be submerged. The excess coating in this instance is shaken or thrown back into the bowl. This detail is not conducive to neatness, besides it necessitates stirring the plaster, another minor detail that is objectionable.

The impression, before it is satisfactory, usually requires three corrections, although four and even five trials are not unusual. If, however, after a greater number of attempts it is apparent that there are corrections still to be made, the plaster may be broken away from the modeling compound tray and the more intimate knowledge of the requirements of the individual case, used to advantage in the successive steps.

These instructions apply in every detail to taking an impression of the lower, with one exception, namely, the patient is required to hold the mouth open and extend the tongue. The tongue in this position prevents the plaster from encroaching upon any part of the lingual attachments that would subsequently move the denture. The lips and cheeks are slightly tense in this position, and if the impression of the lips and cheeks (both upper and lower) are incorporated in the denture the maximum amount of retention, due to valve action, will have been obtained.

It is obvious that any attempt at esthetics by restoring facial contour will defeat retention in direct ratio to the bulk of the restoration. This is equally true of the Green method.

### WILSON METHOD FOR UPPER IMPRESSIONS.

Dr. Wilson describes a method, using a properly shaped and trimmed britannia metal tray with beeswax attached to the posterior border. While the beeswax is still soft an impression is taken at the juncture of the hard and soft palate. This tray, with the beeswax as an integral part, is removed and chilled. The surface of the beeswax is softened over a small flame and the tray returned to the mouth and firm pressure brought to bear upon the tray, just beneath the beeswax. If provision is to be made for the denture to restore the cuspid eminence, beeswax is added in the proper place at this time and the tray returned to the mouth. These additions are then conformed to meet the requirements. Plaster is mixed very thin and placed in this improved tray, the posterior beeswax border being placed in position first, thus preventing the plaster from running down the throat, and firm pressure maintained while the anterior portion of the tray is brought into position with the fingers of the right hand while the thumb and forefinger of the left push the corners of the mouth towards the center of the tray.

DIAGNOSIS OF THE CASE.

Under this heading our contemporary arrives at no scientific determination. The area to be covered by the base is completely ignored in all of the articles which have come to my notice. Is it not reasonable to predict that a base covering an area of 4,000 square millimeters will sustain a greater masticatory stress than one with a corporate area of 3,000 square millimeters? What about the shape of this area within the confines of the base? If the vault is high and the ridges prominent we are assured that the lateral movement of the denture will be practically nil, but the force of occlusion will not be at right angles to its source, an axiomatic truth which physics teaches is essential if we are to expect maximum efficiency. Shall we recken with this factor?

Yes, it is very easy to criticise, but are not the factors of shape and area more important than the muscular attachments and the space between the tuberosities and the coronoid process?

We do not wish to appear facetious or pedantic, but the condition of the saliva, whether it be thin or viscid, plays an important part in the retention of artificial dentures. This ropy condition is remedied by washing the mouth vigorously with a strong solution of alum. If the patient is to have a denture constructed upon a metal base and presents himself with old vulcanite plates, the operator should insist that the impression be taken only when the mouth recovers from the swollen chronic pathological condition usually present. This, as a rule, requires about forty-eight hours and is best accomplished by leaving the artificial substitutes with the dentist.

The age of the patient and his health are important factors, as extreme old age and anemia are conducive to failure. A psychological factor that will aid you in your prognosis is to have some understanding about the compensation that you are to receive and a distinct understanding that one-half of this amount is to be paid when the impressions are taken, the balance when the dentures are inserted. It is well to explain ofttimes, in this connection, that it is less difficult for the patient to wear his own dentures than those owned by the dentist. It is also just and right that a contract be entered into whereby the patient may try the dentures for sixty days after completion (this does not imply from the day they are inserted, as the day of completion may be several days later), and in the event they prove unsatisfactory, four-fifths of all money paid is to be cheerfully refunded.

The most satisfactory results accrue to both patient and dentist *always* when the temptation to substitute the old, comfortable dentures is eliminated by the latter reposing idly in the dentist's lower cabinet drawer.—*Western Dental Journal.*

